# Impact of *Helicobacter pylori* Eradication on Inflammatory Bowel Disease Onset and Disease Activity: To Eradicate or Not to Eradicate?

**DOI:** 10.3390/diseases12080179

**Published:** 2024-08-08

**Authors:** Antonietta Gerarda Gravina, Raffaele Pellegrino, Veronica Iascone, Giovanna Palladino, Alessandro Federico, Rocco Maurizio Zagari

**Affiliations:** 1 Hepatogastroenterology Division, Department of Precision Medicine, University of Campania Luigi Vanvitelli, 80138 Naples, Italy; 2 Department of Medical and Surgical Sciences, University of Bologna, 40138 Bologna, Italy; 3 Esophagus and Stomach Organic Diseases Unit, IRCSS Azienda Ospedaliero-Universitaria di Bologna, 40138 Bologna, Italy

**Keywords:** *Helicobacter pylori*, inflammatory bowel disease, eradication, antibiotics, disease activity

## Abstract

*Helicobacter pylori* infection has significant epidemiological relevance due to the carcinogenic nature of this bacterium, which is potentially associated with cancer. When detected, it should ideally be eradicated using a treatment that currently involves a combination of gastric acid suppressors and multiple antibiotics. However, this treatment raises questions regarding efficacy and safety profiles in patients with specific comorbidities, including inflammatory bowel diseases (IBD). Eradication therapy for *H. pylori* includes components associated with adverse gastrointestinal events, such as *Clostridioides difficile* colitis. This necessitates quantifying this risk through dedicated studies to determine whether this antimicrobial treatment could be significantly associated with IBD relapse or exacerbation of pre-existing IBD, as well as whether it could potentially lead to the de novo onset of IBD. Although the available evidence is reassuring about the safety of eradication therapy in patients with IBD, it is limited, and there are no specific recommendations for this particular situation in the leading international IBD and *H. pylori* guidelines. Therefore, studies need to evaluate the efficacy and safety profiles of the available antimicrobial regimens for *H. pylori* eradication in patients with IBD, both in clinical trial settings and in real-life studies.

## 1. Introduction

*Helicobacter pylori* infection represents a significant global health concern, given its high prevalence in the general population and its association with gastric malignancies [1]. It has been estimated in a comprehensive meta-analysis conducted in 2017 that approximately 4.4 billion individuals are affected by this infection, with a significant international variation; *H. pylori* prevalence rates range from 18.9% in Switzerland to 87.7% in Nigeria [2]. In addition, *H. pylori* is a class I carcinogen agent, according to the International Agency for Research on Cancer, linked to gastric malignancies [3].

As a result, the spontaneous categorical imperative arises to identify the infection in risk conditions (starting with uncomplicated dyspepsia) and treat it appropriately, verifying its eradication as international guidelines recommend [4,5,6]. However, eradicating this bacterium, which frequently experiences antibiotic resistance phenomena, relies on antibiotic therapies that are often particularly complex, involving the combination of proton pump inhibitors (PPIs) with various antibiotics (primarily macrolides, fluoroquinolones, and beta-lactams).

*H. pylori* infection does not exclusively affect the gastro-duodenal microenvironment; rather, it extends its impact to other extra-digestive organs, giving rise to various described extra-gastric manifestations [7]. Haematological manifestations, such as vitamin B_12_ deficiency anaemia, are reported in *H. pylori* infection [8]. When predominantly affecting the gastric body, this infection can damage intrinsic factor-producing cells, thereby exacerbating this phenomenon [9]. Moreover, *H. pylori* can sequester iron from host proteins (e.g., transferrin and lactoferrin) [10]. By increasing the stomach pH, particularly in chronic gastritis affecting the gastric body, *H. pylori* can interfere with the conversion of dietary iron from Fe^3+^ to Fe^2+^, leading to reduced iron absorption and, consequently, the development of iron deficiency anaemia [10]. Finally, molecular mimicry between the surface glycoproteins of platelets and certain amino acid sequences of *H. pylori* virulence factors could stimulate the development of primary immune thrombocytopenia [11,12]. *H. pylori* infection can also affect the skin, being associated with conditions such as rosacea [13], chronic urticaria [14], psoriasis [15], alopecia [16], and autoimmune blistering disorders [17]. As if that were not enough, associations have also been described with diabetes mellitus [18], dyslipidaemia [8], ischaemic stroke [19], Alzheimer’s disease [20], Parkinson’s disease [21], and Guillain–Barré syndrome [22].

All of this underscores that this infection, even before examining its relationships with IBD, should not be mistakenly interpreted as an organ-specific infection. Instead, it potentially systematises, affecting various organs and systems with pathophysiological mechanisms that are not yet fully understood.

The administration of antibiotics may potentially exacerbate or rekindle underlying diseases (particularly when in a state of remission at baseline), such as inflammatory bowel disease (IBD). Inflammatory bowel disease (IBD), primarily encompassing Crohn’s disease (CD) and ulcerative colitis (UC), is an immune-mediated condition giving rise to chronic, relapsing-remitting inflammation of the gastrointestinal tract [23]. The pathogenesis of these diseases also involves intestinal microbiota alterations, a primary adverse effect of antibiotic therapies [23]. Furthermore, antibiotic therapy can contribute to the emergence of dysbiosis-triggered infectious phenomena in IBD, such as *Clostridioides difficile* infection [24]. This infection poses a dual risk factor, being associated with IBD independently and representing one of the leading causes of antibiotic-associated diarrhoea [25,26].

The potential interplay between these two clinical entities varies significantly. Indeed, it appears that *H. pylori* is linked to an exacerbation of the anxious-depressive state [27,28], which, incidentally, serves as a trigger for IBD [29]. Moreover, anxious-depressive states can persist even in conditions of clinical remission of IBD [30]. Nevertheless, *H. pylori* may play a role in the pathogenesis of insulin resistance [31], and it is known that diabetes can negatively impact IBD [32]. These are just a few examples that illustrate the countless implications that can arise from the interaction between *H. pylori* and IBD.

The epidemiological relationship between IBD and *H. pylori* is supported by population genome-wide association studies [33] identifying a certain association between these clinical–pathological entities. However, this relationship is far from being elucidated, being the outcome of a complex epidemiological puzzle. Indeed, *H. pylori* infection may exert a protective effect against the development of IBD [34]. Seropositivity for *H. pylori* virulence factor cytotoxin-associated gene-A (CagA) seems to be associated with a reduced risk of IBD with an odds ratio of 0.26, particularly for CD rather than UC [35].

The protective effect of *H. pylori* against IBD has also been assessed in animal studies. This potential role was highlighted through the intragastric administration of 20 micrograms of endotoxin-free *H. pylori* DNA (derived from the genomes of *H. pylori* SS1, J99, and 26695) to mice with experimental colitis (C57/BL6 mice) induced by 3% dextran sodium sulphate. The mice experienced a significant improvement in experimental colitis, as measured by the disease activity score and the assessment of faecal bleeding [36]. Similarly, other *H. pylori*-derived peptides, such as Hp (2–20), improved experimental colitis in mouse models [37].

Moreover, in addition to extra-digestive manifestations, *H. pylori* infection can affect other digestive non-IBD areas. It can reduce acid secretion in body-predominant forms of chronic gastritis and is inversely associated with gastroesophageal reflux disease [38,39,40]. Additionally, it may be associated with eosinophilic oesophagitis due to the cytokine shift towards a T helper 1 response mediated by *H. pylori*-induced immune tolerance [41,42,43,44]. Finally, irritable bowel syndrome is also associated with this infection [45].

Beyond all of these considerations, antibiotics, unfortunately, constitute both a risk factor and a trigger for the onset of IBD [46]. Moreover, they can potentially exacerbate the disease activity of IBD or lead to its reactivation in the background of disease remission.

Notwithstanding, the significance of this risk in eradicating *H. pylori* in patients with IBD is far from clarified. This review aims to gather targeted studies that have focused on assessing the impact of *H. pylori* eradication as a trigger for the onset of IBD or as a modifying factor of disease activity in patients with pre-existing IBD. Three electronic databases, MEDLINE, EMBASE, and Web of Science, were searched using the following keywords: “Crohn”, “ulcerative colitis”, “inflammatory bowel disease”, “IBD”, “*Helicobacter pylori*”, “*H. pylori*”, “*H. pylori* eradication”, and “antibiotic”. The articles considered for inclusion encompassed research articles, meta-analyses, and case reports, provided they aligned with the objectives of this review.

## 2. *H. pylori* Eradication and Risk of IBD Onset

Several studies suggest a potential role of *H. pylori* eradication therapy in promoting the onset of IBD. In a large cohort study conducted on the National Health Insurance Research Database in Taiwan, one million patients were followed over ten years [47] to assess the risk of developing immune-mediated disorders after *H. pylori* eradication. The authors reported a significant association between *H. pylori* eradication and the development of IBD with a hazard ratio (HR) of 2.15 [95% confidence interval (CI): 1.88–2.46], in particular using triple or quadruple therapy for more than seven days [47].

A retrospective cohort study by Mizukami et al. [48] included the population extracted from the “Japanese nationwide health claims database” from April 2009 to March 2020, encompassing over five million patients undergoing *H. pylori* eradication therapy. The study assessed the incidence of several disorders, including IBD, following *H. pylori* eradication. In this study, the occurrence of both CD and CD exhibited an increase in the three years following eradication, and in particular, the incidence of UC rose for patients aged ≥ 30 years.

Two case reports have reported the onset of CD after eradicating *H. pylori* in young individuals [49]. Nevertheless, another report has also noted the onset of UC in a 72-year-old patient within a few days after the completion of the eradication antibiotic regimen [50]. A similar case has been reported for another 63-year-old patient [51]. Table 1 summarises these case reports.

Among the most recurrent hypotheses in the literature attempting to postulate the mechanism underlying the relationship between the eradication of *H. pylori* infection and the onset of IBD is the interruption that eradication has on the immunomodulatory properties of *H. pylori* [52,53,54]. It is known that long-standing *H. pylori* infection leads to a shift in the phenotype of T helper lymphocytes towards type 2 rather than type 1 (the latter phenotype being more commonly described in patients with CD) [44]. Therefore, eradication could, in other terms, interrupt this shift and promote a sudden increase in T helper 1 cytokines (such as interleukins 4, 5, and 6) [55].

In addition, a potential microbiome-modulating mechanism is evident in the inverse association observed between *H. pylori* and putatively proinflammatory microbial entities (*Bacteroides ovatus*, *Fusobacterium varium*, *Rhodococcus*, and *Sphingomonas*) [56]. Consequently, the immunomodulatory impact of *H. pylori* on the gut microbiota is also a pertinent factor that must be considered.

However, the major challenge in establishing a clear causal link between the eradication of *H. pylori* infection and the de novo onset of IBD is that eradication therapy involves drugs, i.e., antibiotics and PPIs, that are explicitly associated with environmental trigger factors for IBD [46,57,58].

The antibiotics most commonly used in *H. pylori* eradication regimens include clarithromycin, amoxicillin, metronidazole/tinidazole, levofloxacin, and tetracycline [5,6]. These antibiotics are combined with PPIs in various combinations, resulting in different “regimens”, such as triple and quadruple therapies, including bismuth quadruple, concomitant, and sequential therapies [5,6].

However, despite their proven efficacy in eradicating *H. pylori*, these antibiotics have a well-defined broad spectrum of action [59,60,61,62,63]. This inevitably allows these antibiotics to profoundly alter the intestinal microenvironment and gut microbiota, primarily when combined [64,65].

Before the availability of advanced therapies for IBD (i.e., biologic drugs and surgical techniques), antibiotics were considered as potential therapeutic agents in IBD, demonstrating a diverse spectrum of therapeutic potential in inducing disease remission [66]. However, given their safety profile, it is evident that current guidelines do not advocate their use in the therapeutic sequencing of IBD [67,68,69]. On the other hand, it is well established that the use of antibiotic therapies with broad-spectrum antimicrobial coverage carries an increased risk of IBD onset. This has been reported, for instance, by an extensive North European case-control study involving over twenty thousand patients [70].

PPIs, which are a cornerstone in *H. pylori* eradication regimens, can also increase the risk of IBD. This has been observed, for instance, in a prospective cohort with an estimated HR of 1.42 [71] and in a large population study over a two-year time frame from the initiation of PPIs with a comparable HR of 1.34 [72]. Nevertheless, in some settings, it has been reported that the concurrent use of PPIs with certain therapies (i.e., vedolizumab) may counteract their therapeutic impact in achieving clinical response [73]. In addition, the use of PPIs in patients with IBD may also entail a tripled risk of enteric infections [74].

## 3. *H. pylori* Eradication and Recurrence or Exacerbation of IBD

Numerous studies have investigated the impact of *H. pylori* eradication on the disease activity of underlying IBD. It has been postulated and extensively debated whether patients with IBD may experience a deterioration in disease activity following antibiotic therapy for *H. pylori* eradication.

A recent meta-analysis showed that individuals with IBD faced a 1.41-fold increased risk of disease relapse after *H. pylori* eradication [75]. On the other hand, Rosania et al. [76] did not observe a significant rate of recurrence following *H. pylori* eradication in a sample comprising over a hundred patients with IBD.

In a retrospective cohort study involving over 400 IBD patients in Japan, the use of standard amoxicillin-based triple therapy for *H. pylori* eradication did not result in a higher rate of worsening disease activity at two and six months compared to the non-eradication control group [77]. 

Other studies deliberately excluded patients presenting with active IBD at baseline, as exemplified by Lahat et al. [78], who examined the impact of eradication therapy on clinical disease activity using the CD activity index (CDAI) and biochemical markers (C-reactive protein and faecal calprotectin) in CD patients. Importantly, no discernible and statistically significant alterations in these parameters were documented up to 8 weeks post-antibiotic therapy. Nevertheless, it is crucial to acknowledge the limited robustness of conclusions drawn from this investigation, given its notably restricted cohort size—only six patients identified as *H. pylori* positive within an initial pool of 56 participants.

However, significant limitations exist in interpreting this data. Dedicated trials assessing the clinical and endoscopic disease activity of IBD with validated outcomes before and after *H. pylori* eradication therapy are lacking. Table 2 summarises the key findings from studies on this matter.

Another concept to consider, which is indirectly associated with IBD disease activity and its relationship with *H. pylori* infection, is the etiological convergence on the pathogenesis of anaemia. It is indeed known, as previously stated, that *H. pylori* can cause various forms of anaemia, the most significant being vitamin B_12_ and iron deficiency anaemia [8]. Conversely, IBD can also cause these same forms of anaemia. In detail, IBD can cause iron deficiency anaemia due to a bimodal loss of iron, both from gastrointestinal bleeding and reduced absorption, especially in patients with UC [80]. Vitamin B_12_ or folate deficiency anaemia can occur, particularly in patients with CD, due to malabsorption of these elements and significant intestinal resections [80]. For these reasons, anaemia is currently considered one of the most common and widespread extra-intestinal manifestations associated with IBD [81]. It requires careful monitoring throughout the IBD natural history and prompt treatment, including parenteral iron supplementation when haemoglobin levels are below ten g/dL [81]. This prompts a reflection on the fact that *H. pylori*, when associated with these haematological manifestations, especially in the context of active IBD, can further exacerbate them.

## 4. *H. pylori* Eradication and Osteoprotegerin Levels in Patients with IBD

Osteoprotegerin, a molecule secreted by osteoblasts, belongs to the tumour necrosis factor receptor superfamily (i.e., TNFR) [82]. It assumes a multifaceted role in bone metabolism through the orchestration of inflammatory processes and is implicated in tumorigenesis [82]. Serving as an inhibitory agent, it exerts regulatory control over osteoclastogenesis within the skeletal framework [83]. Osteoprotegerin has been consistently proposed as a non-invasive marker in IBD, as its levels seem to correlate with disease activity [82]. On the contrary, the connection between osteoprotegerin and, more broadly, bone metabolism in the context of *H. pylori* infection appears less apparent [84,85].

A case-control study assessed the effect of sequential eradication therapy for *H. pylori* on osteoprotegerin serum levels and alkaline phosphatase (bone isoform) [86]. This study showed that the levels of these indicators of bone metabolism remained unaffected by the eradication therapy [86]. Within the constraints of results derived from a single study and the ongoing elucidation of the intricate relationship between *H. pylori* infection and osteoprotegerin, this finding provides reassurance regarding the effects of eradication therapy on this protective indicator of bone metabolism.

## 5. The Risk of Post-Antibiotic- and PPI-Related *C. difficile*: The Third Party to Consider

*C. difficile* infection recognises the use of antibiotics, particularly broad-spectrum antibiotics, as the most significant risk factor [24]. Among these, those most commonly associated with *C. difficile* infection include the antibiotics used in eradicating *H. pylori* infection, such as penicillins and fluoroquinolones [87,88,89]. For instance, Brown et al. [90], in a large longitudinal cohort study, identified a relative risk of 2.21 (95% confidence interval 1.67–3.08) between the use of amoxicillin and the risk of *C. difficile* infection. Amoxicillin is a pertinent example in this context, as it is a leading antibiotic in eradicating *H. pylori* infection and is included in various regimens. These regimens include concomitant therapy, sequential therapy, classical triple therapy with clarithromycin, triple therapy with levofloxacin, and advanced therapies with rifabutin and high-dose dual PPI therapy [5,6].

In addition, the use of PPIs alone, without considering the combination *H. pylori* eradication regimens with antibiotics, is already associated with an increased risk of enteric infections, including *C. difficile*. This is confirmed by a large cohort study that identified the use of PPIs as a variable significantly associated with an increased relative risk (2.1) for *C. difficile* infection [91]. Moreover, this has been further validated by a large meta-analysis of 300,000 patients, where the use of PPIs was associated with a 65% increase in the risk of developing *C. difficile*-associated diarrhoea [92].

The digestive antibiotics-driven damage caused by *C. difficile* is primarily mediated through toxin-driven pathogenesis [93]. Diarrhoea and pseudomembranous colitis are manifestations resulting from the action of toxin A (i.e., clostridial glycosylation exotoxins), which is an enterotoxin, and toxin B (i.e., a cytotoxin) [93]. Colonocytes internalise these toxins and, by inactivating a Rho-mediated signalling pathway, cause damage to tight junctions and recruit neutrophils, creating a pro-inflammatory microenvironment and disrupting the integrity of the colonic epithelial barrier [93].

Conversely, the relationship between IBD and *C. difficile* is particularly complex. IBD can contribute to its pathogenesis through intestinal dysbiosis [23], and in turn, dysbiosis can be caused by IBD, thereby increasing the risk of *C. difficile* infection.

Nonetheless, a meta-analysis has identified several IBD-specific risk factors for *C. difficile* infection in this population, including colonic involvement in CD and the use of biologics [94]. Moreover, this infection is a clear risk factor for colectomy in patients with IBD (especially those with UC), nearly doubling the risk when it is present [95].

It is not surprising, therefore, that another meta-analysis, which included over 40,000 IBD patients with *C. difficile* infection compared to over one million control IBD patients, demonstrated an increase in mortality (odds ratio 4.39) in UC patients affected by this infection [96].

Therefore, in balancing the Hamlet-like dilemma between eradicating and not eradicating *H. pylori* infection, it is also essential to consider the variable of *C. difficile*.

Among the few studies that have examined the relationship between *H. pylori* eradication and the subsequent risk of *C. difficile* infection, Kumar et al. [97] conducted a large retrospective cohort study that included nearly 40,000 patients (predominantly older than sixty years and almost entirely male) who tested positive for *H. pylori*. The study assessed the rate of *C. difficile* infection within 3 months of identifying *H. pylori* infection by constructing a multivariate logistic regression on this sample to evaluate, among the included variables, the eradication therapy for *H. pylori*. Of the initial sample, 74.8% were treated for *H. pylori*, with 0.74% subsequently developing a *C. difficile* infection. There was no clear significant association, as confirmation of successful eradication did not increase the risk of subsequent *C. difficile* infection (odds ratio 1.49, 95% confidence interval 0.67–3.29) [97]. Another large retrospective study involving 11,457 patients with *H. pylori* observed an equally minimal rate of *C. difficile* infection (i.e., 0.21%) in patients who underwent eradication therapy [98]. However, these data, though extensive and reassuring, are derived from the general population. Currently, we do not have data specifically directly applicable to IBD populations.

This should probably encourage clinicians eradicating *H. pylori* infection in patients with IBD to carefully monitor for any post-eradication diarrhoea that is not related to IBD. This requires conducting a differential diagnosis [99,100], which, although challenging, is necessary to distinguish between a possible *C. difficile* infection and an IBD relapse.

In addition, probiotic supplementation alongside *H. pylori* eradication therapy in IBD patients could likely be even more desirable given these considerations [5,6,101].

In summary, *C. difficile* infection is a highly relevant complication associated with *H. pylori* eradication therapy in patients with IBD and must be carefully considered when choosing to eradicate the bacterium for chemoprevention of gastric cancer in IBD patients. This is especially important given the exceedingly rare but potentially fatal cases of complicated *C. difficile* infection [102,103,104].

As highlighted by these case reports (Table 3), many of the infections, though rare, have exposed patients to severe complications, including surgery. It is well known that patients with IBD have highly variable post-surgical outcomes, which are delicately influenced by various pre-operative factors, including nutrition and the concurrent use of steroids or biological drugs. Nevertheless, surgery has a dual aspect. Although it can be definitive in UC, it must be performed in specialised centres where restorative proctocolectomy with ileal pouch is the desirable choice (although not always possible, especially in a single operation) [105]. On the other hand, in CD, surgery does not lead to a cure and often necessitates prophylaxis to prevent post-surgical recurrence.

Consequently, exposing patients with IBD to emergency surgery due to a complicated *C. difficile* superinfection is a severe complication. Therefore, clinicians must closely monitor the eradication of *H. pylori* in patients with active IBD, those who are fragile, and those with non-IBD and IBD-related risk factors associated with an increased risk of *C. difficile* infection. It is probably advisable to initiate *H. pylori* eradication when modifiable risk factors have been ideally removed. However, the lack of guidelines and evidence on how to proceed in these cases is significant and requires efforts from the scientific community in this regard.

## 6. Conclusions

In conclusion, evidence indicates that, despite the significant oncological preventive benefits of eradicating *H. pylori*, there is an epidemiological downside concerning the potential de novo onset of IBD. Additionally, a nuanced examination of the impact of *H. pylori* eradication regimens on IBD activity is crucial, requiring dedicated studies utilising validated tools [106] for subjective clinical assessment and, importantly, objective measures such as biochemical and endoscopic evaluations.

The urgent and pertinent need for “understanding how to proceed” in the management of *H. pylori* infection in patients with IBD is particularly pronounced, especially in geographical contexts where mass screening for *H. pylori* infection is justified to control morbidity and mortality for gastric cancer [107,108,109].

Clear-cut criteria are essential for guiding IBD specialists in distinguishing genuine exacerbation or worsening of IBD post-eradication therapy from routine adverse events associated with antibiotic or acid-suppressive treatments. Conversely, determining whether abstaining from *H. pylori* eradication in IBD patients is justifiable poses a formidable challenge.

These unmet needs underscore the need for studies focusing specifically on the IBD population or post hoc analyses of extensive studies conducted for the general population to gather evidence relevant to various IBD subgroups.

## Figures and Tables

**Table 1:** Case reports related to the onset of inflammatory bowel disease (IBD) after the eradication treatment of *Helicobacter pylori* (*H. pylori*) infection.

Gender	Age	Comorbidity	*H. pylori* Onset	*H. pylori* Treatment	IBD	Ref
Male	28	Liver capillary haemangioma	Dyspepsia	Omeprazole and amoxicillin	CD (L1)	[49]
Male	34	None	Epigastric pain and vomiting	Esomeprazole, amoxicillin, and clarithromycin	CD (L1)	[52]
Female	39	Previous duodenal ulcer (3 years before being treated with triple therapy)	Epigastric pain	Esomeprazole, amoxicillin, and clarithromycin	CD (L2)	[52]
Female	72	Chronic gastritis and gastroesophageal reflux disease, osteoporosis, thyroidectomy (goitre and Hashimoto’s thyroiditis), arterial hypertension, Reinke’s syndrome	Dyspepsia	Rabeprazole, clarithromycin, metronidazole, and probiotic supplementation	UC (E3)	[50]
Male	63	Vertigo, arterial hypertension	None (gastric cancer screening)	Lansoprazole, amoxicillin, clarithromycin	UC (proctosigmoiditis)	[51]

CD: Crohn’s disease; UC: ulcerative colitis; L1: ileal CD; L2: colonic CD; E3: pancolitis.

**Table 2:** Studies investigating the effects of *Helicobacter pylori* (*H. pylori*) eradication on the disease activity of underlying Crohn’s disease (CD) and/or ulcerative colitis (UC).

First Name (Year)	N (IBD)	Country	Main *H. pylori* Eradication Treatment Employed	Main Result	Ref
Lahat et al. (2017)	6/56 (CD)	Israel	Sequential therapy	In the subgroup of *H. pylori*-positive patients (N = 6), no significant variations in clinical activity (CDAI) and biochemical (C-reactive protein and faecal calprotectin) markers of disease were observed	[78]
Rosania et al. (2018)	127 (IBD)	Germany	Clarithromycin-based triple therapy; quadruple therapy with Pylera^®^ and omeprazole	Only 3% of the sample experienced a recurrence after *H. pylori* eradication	[76]
Shinzaki et al. (2018)	429 (IBD)	Japan	Amoxicillin-based triple therapy	The exacerbation rate at two months (8.3%) and six months (11.8%) was not significantly different from the control group.	[77]
Fujita et al. (2021)	1 (UC)	Japan	Amoxicillin-based triple therapy	UC relapse after *H. pylori* eradication.	[79]

IBD: Inflammatory bowel disease.

**Table 3:** Relevant clinical cases reporting *Clostridioides difficile* infections associated with eradicating therapy for *Helicobacter pylori* infection.

Gender	Age	Comorbidity	*H. pylori* Treatment ^1^	*C. difficile* Infection Relevant Data	Outcome	Ref
Male	74	Previous lymphoproliferative disease, dyslipidaemia, hyperuricaemia	Vonoprazan, probiotics, amoxicillin (750 mg/q12 h), sitafloxacin (200 mg/q12 h)	Fulminant colitis, cardiopulmonary arrest followed by emergency colonic resection after resuscitation, and severe metabolic acidosis post-surgery (death following a second cardiopulmonary arrest).	Death	[102]
Female	70	Hypertension, colonic diverticulosis	7 days: pantoprazole 40 mg/24 h, clarithromycin 1 g/24 h, amoxicillin 2 g/24 h	Failure to oral metronidazol 500 mg/q8 h followed by response to vancomycin 125 mg/q6 h.	Resolution	[104]
Female	51	None	7 days: pantoprazole 40 mg/24 h, clarithromycin 1 g/24 h, amoxicillin 2 g/24 h	Response to oral vancomycin 125 mg/q6 h	Resolution	[104]
Female	68	None	Vonoprazan, amoxicillin, clarithromycin	Failure to ceftriaxone followed by septic shock, disseminated intravascular coagulation. An emergency colonic resection followed a trial with metronidazole and vancomycin	Resolution	[103]

^1^ If the dosages of some of the combination drugs used for the eradication of *H. pylori* infection are not mentioned, it is because they are not available/specified in the original work. q12 h: every 12 h; q8 h: every 8 h; q6 h: every 6 h.

## Data Availability

No new data were created or analyzed in this study. Data sharing is not applicable to this article.
